# Synthesis and characterization of RF-sputtered ZnTe/Cu_2−_*_x_*Te thin films for solar cell applications

**DOI:** 10.3762/bjnano.17.69

**Published:** 2026-07-31

**Authors:** Gerardo Arreola Jardón, Susana Meraz Dávila, Claudia Elena Pérez García, Aime Margarita Gutiérrez Peralta, Sergio Joaquín Jiménez Sandoval

**Affiliations:** 1 Universidad Politécnica de Santa Rosa Jáuregui, Querétaro México, Carretera Federal 57 QRO- SLP Km. 31 + 150, Santa Rosa Jáuregui, 76220 Querétaro, Qro, México; 2 Facultad de Ciencias Químicas, Universidad Autónoma de Querétaro, Cerro de las Campanas, C.P. 76010, Santiago de Querétaro, Qro, México; 3 Centro de Investigación y de Estudios Avanzados del Instituto Politécnico Nacional Unidad Querétaro, Libramiento Norponiente 2000, 76230 Querétaro, Qro, México

**Keywords:** back contact materials, Cu_2−_*_x_*Te phases, phase segregation, RF magnetron sputtering, ZnTe thin films

## Abstract

The incorporation of copper and oxygen into zinc telluride (ZnTe) thin films deposited by radio-frequency magnetron sputtering from a single ZnTe–CuO composite target was investigated. The nominal Cu and O concentrations ranged from 3 to 13 atom %, and films were grown at substrate temperatures of 300 and 350 °C. Energy-dispersive X-ray spectroscopy confirmed controlled compositional transfer from the target to the films. X-ray diffraction analysis revealed that all films are polycrystalline, exhibiting a coexistence of zinc blende and wurtzite ZnTe phases. Low dopant concentrations produced only minor lattice modifications, while higher Cu and O contents promoted the formation of Cu_2−_*_x_*Te secondary phases, as confirmed by Raman spectroscopy, grazing-incidence X-ray diffraction and scanning electron microscopy. Optical measurements showed a significant reduction in infrared transmittance with increasing Cu concentration, attributed to the metallic-like absorption of Cu_2−_*_x_*Te phases. Electrical characterization revealed a transition from semiconducting to highly conductive behavior with resistivity decreasing from approximately 10^2^ to 10^−2^ Ω·cm for films grown at 300 °C and from 10^1^ to 10^−3^ Ω·cm for films grown at 350 °C. Simultaneously, the carrier concentration increased from approximately 10^17^ to 10^21^ cm^−3^ and the mobility from 10^−1^ to 10^1^ cm^2^·V^−1^·s^−1^. These results indicate that structural, optical, and electrical properties of ZnTe are primarily influenced by Cu incorporation and the formation of conductive Cu-rich secondary phases, yielding a biphasic system composed of semiconducting ZnTe and conductive Cu-rich telluride phases. These material properties suggest potential relevance for future studies of back-contact materials in CdTe-based solar cells.

## Introduction

CdTe solar cells remain one of the leading thin-film photovoltaic technologies due to the near-optimal direct bandgap of CdTe (≈1.45 eV) and its high absorption coefficient (>10^4^ cm^−1^), which allows for the absorption of nearly 99% of incident sunlight within a thickness of approximately 1 µm. These intrinsic optoelectronic properties enable efficient light harvesting while reducing material consumption compared to conventional crystalline silicon technologies [1–2]. As a result, CdTe-based modules have achieved certified power conversion efficiencies exceeding 21% at the industrial level, demonstrating the technological maturity of this material system [3].

Despite these advances, further improvements in device efficiency, long-term stability, and cost reduction remain critical challenges for large-scale application [4]. One of the most persistent limitations in CdTe solar cells is the formation of a stable back contact with low resistance. The high work function of CdTe (≈5.7 eV) and its relatively high surface resistivity hinder the formation of an ideal ohmic contact with conventional metals. As a consequence, Schottky barrier formation at the CdTe/metal interface may occur, leading to rollover effects and device degradation [1].

Copper-containing compounds have been widely employed to mitigate this issue as Cu acts as an acceptor dopant and promotes the formation of a hole region at the CdTe surface, reducing the barrier height. However, uncontrolled Cu diffusion through grain boundaries and toward the CdS/CdTe junction has been associated with long-term instability and performance degradation. Therefore, controlling Cu incorporation while minimizing its diffusion remains an unresolved scientific and technological challenge [5].

Among the proposed strategies, Cu*_x_*Te thin films have been introduced as interfacial layers to improve back contact properties. Nevertheless, these films are typically grown at relatively high temperatures (250–300 °C), conditions that may still promote Cu diffusion into the CdTe absorber. Furthermore, the complex polymorphic nature of the Cu*_x_*Te system and the difficulty in phase control may result in inconsistent electrical behavior and reduced reproducibility. Similarly, ZnTe:Cu thin films deposited by RF sputtering have been investigated as alternative back contact materials. Although ZnTe exhibits intrinsic p-type conductivity and a wide bandgap (2.24 eV), its relatively low conductivity and the diffusion of Cu during growth remain limiting factors for device performance [6–8].

ZnTe has attracted considerable attention not only as a photovoltaic material but also as a contact and buffer layer in thin-film solar cells due to its favorable band alignment with CdTe and its chemical compatibility [9–16]. However, improving its electrical properties without inducing excessive Cu diffusion continues to be a key challenge.

In recent years, composite and dual-doping approaches have emerged as promising alternatives to tailor structural, optical, and electrical properties in II–VI semiconductors. In our research group, composite systems such as CdTe:Cu_2−_*_x_*Te and CdSe:Cu_2−_*_x_*Te have been successfully developed, demonstrating that controlled incorporation of Cu and O through RF sputtering enables modification of the base semiconductor properties while reducing uncontrolled Cu migration [17–22]. These studies showed that composite growth strategies can significantly decrease resistivity in materials such as CdTe, CdSe, and ZnTe, without evidence of excessive Cu^2+^ formation, thereby favoring improved electrical stability.

Previous studies on Cu-doped ZnTe thin films have mainly focused on the influence of Cu incorporation on crystallinity, optical bandgap, and electrical transport properties [23–25]. Similarly, investigations on Cu-rich telluride systems have demonstrated the strong impact of secondary conductive phases on the electrical response of chalcogenide materials [26–28]. However, a systematic study of ZnTe-based films deposited from a single ZnTe–CuO composite target, including the combined effects of composition and substrate temperature on phase evolution, microstructure, and functional properties, remains limited. In particular, the relationship between Cu-induced phase segregation, structural evolution, and the resulting electrical transition has not been fully established.

Based on this approach, the present work investigates ZnTe-based films deposited from a ZnTe–CuO composite target over a broad nominal composition range (3–13 atom %) and at two substrate temperatures (300 and 350 °C). The objective is to establish the correlation between phase evolution, microstructural changes, and the resulting optical and electrical properties, as well as to determine whether simultaneous Cu and O incorporation leads to the formation of a homogeneous quaternary phase or to phase segregation phenomena. This strategy provides insight into the mechanisms governing Cu incorporation, phase segregation, and conductivity evolution in ZnTe-based films. In particular, it enables the identification of the transition from predominantly substitutional Cu incorporation at low concentrations to conductivity governed by Cu-rich secondary phases at higher concentrations, establishing a direct relationship between phase evolution and electrical transport. These findings contribute to the understanding and development of ZnTe-derived materials for back-contact applications in CdTe solar cells.

## Experimental

The films were grown by radio-frequency (RF) magnetron sputtering on a glass substrate and n-type silicon wafers with (100) orientation. Silicon substrates were chosen to avoid interference from oxygen signals originating from the glass substrate during compositional analysis and to ensure reliable chemical quantification.

Targets of two inches diameter were prepared by cold pressing at room temperature using a mixture of ZnTe (Aldrich, 99.99%) and CuO (Plasma Science 99.99%) powders. The powder mixtures were homogenized prior to pressing. The targets were prepared with nominal Cu and O concentrations of 0, 3, 8, 10, and 13 atom %.

During film growth, the Ar flow in the sputtering chamber was maintained at 11 sccm, and an RF power of 50 W was applied to the target. The target-to-substrate distance was 8 cm, and the substrate temperatures were 300 and 350 °C. These substrate temperatures were chosen to prevent the formation of the amorphous phases, which have been reported to occur at temperatures below 250 °C in RF sputtering systems [20]. The base pressure prior to deposition was ≈10^−5^ Torr and the working pressure during growth was maintained at approximately ≈2 × 10^−3^ Torr. The targets were pre-sputtered for 5 min to remove surface contaminants, and the deposition time was fixed at 60 min for all samples.

### Structural and chemical characterization

The chemical composition of the films grown on silicon substrates was determined by energy dispersive X-ray spectroscopy (EDS) using a scanning electron microscope (XL30, ESEM, Phillips) equipped with EDX (EDAX) and an electronic microanalysis probe (EPMA) JXA-8530F. Multiple measurements were performed on different regions of each sample, and the reported values correspond to averaged compositions obtained using the ZAF correction method.

X-ray diffraction (XRD) patterns were acquired using a D/Max-2100 (Rigaku) diffractometer with Co Kα radiation (λ = 1.7889 Å). Raman spectra were obtained in a micro-Raman system (Labram, Dilor) with a 20 mW He–Ne laser (λ = 632.8 nm) focused through a 50× objective, resulting in a spot size of approximately 2 µm. Neutral density filters were employed to minimize local heating effects and avoid structural modifications during measurement.

Normal-incidence transmittance spectra were recorded in the wavelength range of 400–2500 nm using a Cary 5000 spectrophotometer (Agilent Technologies). Finally, the charge transport parameters (carrier concentration, mobility, and resistivity) were determined by Hall effect measurements using the Van der Pauw configuration at room temperature.

## Results and Discussion

### Chemical composition

**Figure 1:**
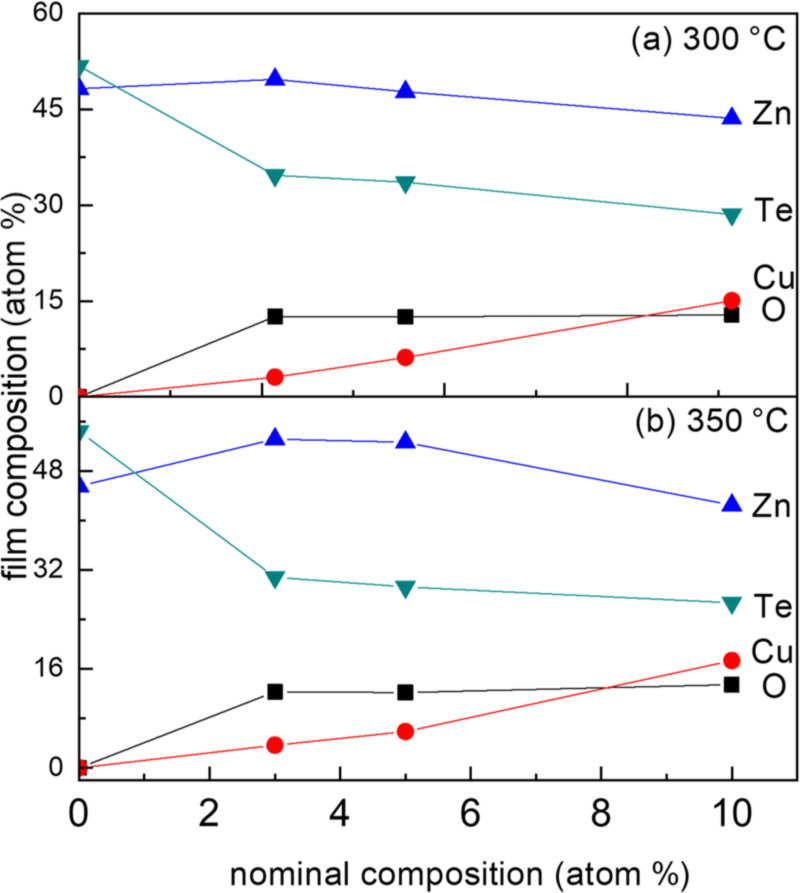
Chemical composition of CuZnTeO films as a function of the nominal Cu and O concentration in target for substrate temperatures of (a) 300 °C and (b) 350 °C.

The chemical composition of the CuZnTeO films was determined by EDS and is presented in Figure 1 as a function of the nominal Cu and O concentration in the targets for substrate temperatures of 300 °C (Figure 1a) and 350 °C (Figure 1b). As expected, the Cu content in the films shows a monotonic increase with increasing nominal Cu composition in the target, indicating a controlled incorporation process. In particular, a nearly linear dependence between nominal and measured Cu concentration is observed, suggesting good compositional transfer from target to films under selected sputtering conditions.

Additionally, an increase in the [Zn]/[Te] ratio is observed in films grown at 350 °C compared to those grown at 300 °C. This behavior is attributed to enhanced Te re-evaporation at higher substrate temperatures, due to the relatively high vapor pressured of Te during growth. Such temperature-dependent deviations from stoichiometry have been previously reported in ZnTe thin films [29–30]. It is worth noting that ZnTe films with a near-stoichiometric [Zn]/[Te] ≈ 1 ratio are typically obtained within the substrate temperature range of 300–350 °C, which is consistent with the growth window selected in this work.

Another relevant observation is that the ratio [Cu + Zn]/[Te + O] remains approximately unity for most samples. This may suggest that Cu and O are primarily incorporated through substitutional mechanisms at Zn and Te lattice sites, respectively, rather than forming independent secondary phases at low concentrations. The substitution of O at Te sites is expected to occur when the oxygen concentration is relatively low (typically below 1 atom %). At higher O concentrations, phase segregation may occur [31–32]. Furthermore, Cu incorporation at Zn sites is structurally plausible due to the similar ionic radii and electronic configurations of Cu^2+^ (73 pm) and Zn^2+^ (74 pm) [33], which facilitates substitutional doping without significant lattice distortion.

### Crystalline structure

**Figure 2:**
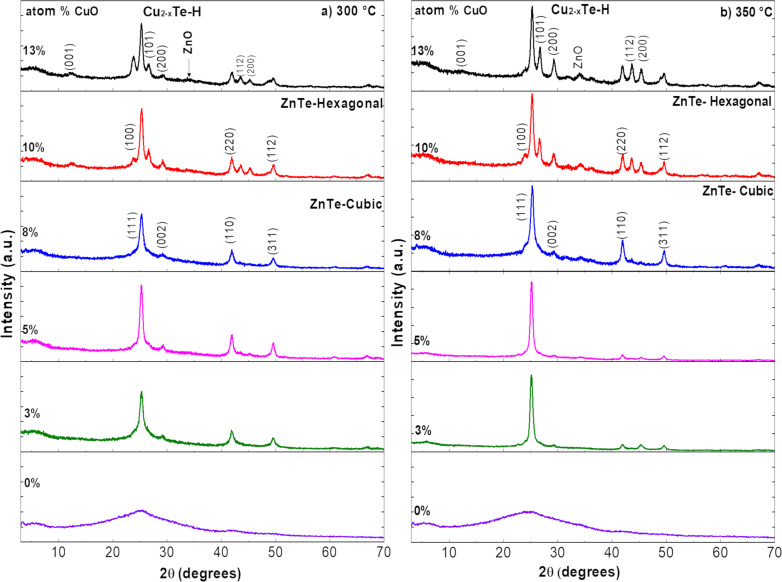
X-ray diffraction patterns of ZnTe and CuZnTeO films as a function of nominal Cu and O concentration at substrate temperatures of (a) 300 °C and (b) 350 °C.

Figure 2 shows the XRD patterns of ZnTe and CuZnTeO films grown on glass substrates as a function of the nominal Cu and O concentration and substrate temperature. In general, ZnTe films grown at substrate temperatures below 300 °C are reported to exhibit amorphous or poorly crystalline structures. However, films deposited at 300 and 350 °C in the present study are clearly polycrystalline, as evidenced by well-defined diffraction peaks.

For films with nominal Cu and O concentrations ranging from 3 to 13 atom %, a dominant diffraction peak is observed at 2θ ≈ 25.29°, along with weaker reflections in the 2θ range of 40–50°. These peaks correspond to a mixture of ZnTe crystalline phases, namely zinc blende [JCPDS 65-5730] and wurtzite [JCPDS 19-1482]. The coexistence of these two polymorphs is commonly reported in sputtered ZnTe films and is associated with growth kinetics and substrate temperature effects.

For films with Cu and O concentrations between 3 to 8 atom %, no significant structural modifications are observed compared to pure ZnTe films reported in the literature [34–35], indicating that low dopant incorporation does not drastically alter the host lattice structure. However, a notable reduction in the full width at half maximum (FWHM) of the main diffraction peak is observed for films containing 3 and 5 atom % Cu and O when the substrate temperature increases from 300 to 350 °C. Specifically, the FWHM decreases from 0.98° to 0.55° (3 atom %) and from 0.58° to 0.51° (5 atom %), respectively. This reduction in FWHM suggests an improvement in crystalline quality and an increase in crystallite size, consistent with thermally enhanced grain growth processes [36]. To quantify this effect, the crystallite size of the ZnTe phase was estimated using the Scherrer equation, considering the main reflections associated with the cubic zinc blende structure. The calculated crystallite sizes ranged from 11.28 to 17.07 nm, confirming the nanocrystalline nature of the films and supporting the enhancement in crystallinity with increasing substrate temperature.

In contrast, films grown with Cu and O concentrations greater than 8 atom % exhibit additional diffraction peaks at 2θ ≈ 26.75° and in the 40–50° range. These reflections are attributed to the formation of Cu_2−_*_x_*Te-type crystalline phases, similarly to those reported in [28]. The emergence of these secondary phases indicates that, above a critical dopant concentration, the solubility limit of Cu and O in the ZnTe lattice is exceeded, leading to phase segregation. In general, the films grown at 350 °C show the same crystalline phases identified at 300 °C (Figure 2a), although with improved peak definition, reflecting enhanced crystallinity at higher substrate temperature.

**Figure 3:**
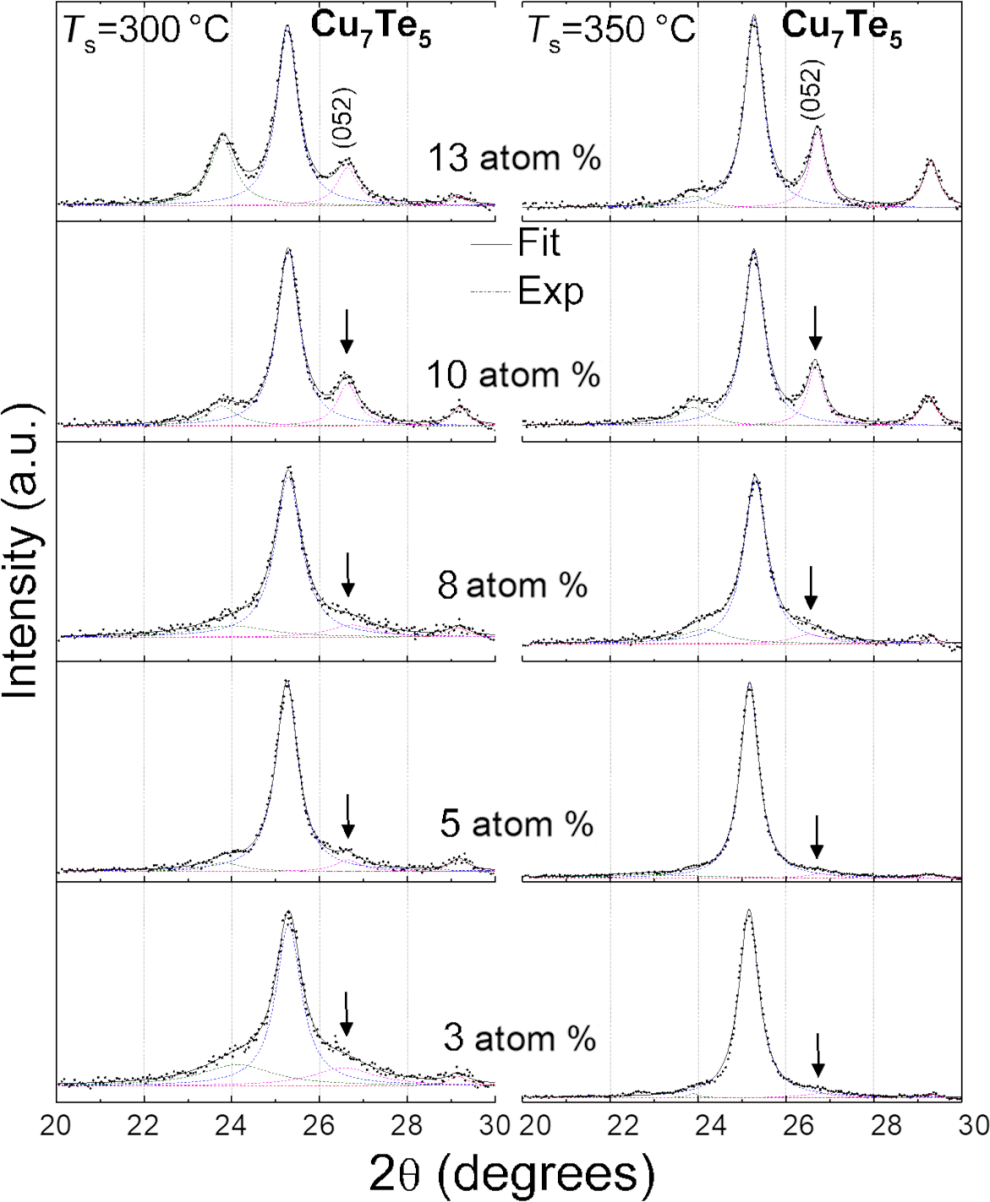
Deconvolution of the main peak the (111) plane of the cubic phase and the (200) plane of the hexagonal phase of ZnTe and CuZnTeO films as a function of the nominal concentration of CuO for substrate temperatures of 300 °C (left) and 350 °C (right).

A more detailed analysis was performed around the main diffraction peak corresponding to the (111) plane of the cubic phase and the (200) plane of the hexagonal phase (Figure 3), in order to gain deeper insight into the structural modifications associated with the presence of Cu and O in the films. The peak deconvolution results for films grown at substrate temperatures of 300 °C and 350 °C are presented in Figure 3.

The deconvoluted peaks were assigned according to the reference patterns of ZnTe and Cu_7_Te_5_. The diffraction contribution centered at approximately 2θ ≈ 25.3° was associated with the ZnTe phase, whereas the reflection observed near 2θ ≈ 26.6° was attributed to the Cu_7_Te_5_ secondary phase. Therefore, the deconvolution analysis was primarily used to evaluate the structural evolution and phase segregation induced by increasing Cu concentration rather than to directly identify oxygen incorporation.

For the film containing 3 atom % Cu and O, the main ZnTe-related peak is located at 2θ ≈ 25.29°. With increasing dopant concentration, a slight shift of this peak is observed, reaching 2θ ≈ 25.26° for 5 atom % and 8 atom %, and shifting to 2θ ≈ 25.31° for 10 atom %. These small variations in peak position indicate minor lattice parameter modifications, consistent with a low degree of structural perturbation of the ZnTe host lattice. Such behavior is compatible with substitutional incorporation of Cu at Zn sites, as previously reported in the literature [23,25,32], where Cu is preferentially incorporated into ZnTe lattice due to the similarity of its ionic radius and electronic configuration with Zn, leading to modifications in crystallinity and lattice-related parameters. In contrast, the oxygen contribution is not directly evidenced by XRD because no oxygen-related crystalline phase was detected. The oxygen present in the films is likely associated with residual oxygen introduced during deposition, post-deposition exposure to air, or growth-related processes, as previously reported for chalcogenide thin films [37], although its specific chemical state cannot be determined from XRD analysis. Therefore, the peak analysis was used to assess the overall structural evolution of the films in the presence of Cu and O rather than to directly identify oxygen incorporation into the ZnTe crystal lattice. The magnitude of the observed peak shift is relatively small, indicating that the overall ZnTe crystal structure is largely preserved within this concentration range. Such subtle peak displacements may be associated with localized strain effects or slight changes in lattice parameters resulting from Cu incorporation at Zn lattice sites.

Additionally, a diffraction peak located at 2θ ≈ 26.60°, corresponding to the (052) plane of the Cu_7_Te_5_ phase (JCPDS 26-1117), is detected in all doped films. The intensity of this reflection increases with both nominal Cu concentration and substrate temperature, indicating progressive formation and growth of the Cu_7_Te_5_ secondary phase. This behavior suggests that, beyond a certain solubility limit, excess Cu promotes phase segregation, leading to the coexistence of ZnTe and Cu_7_Te_5_ phases within the films. The increase in Cu_7_Te_5_ peak intensity with substrate temperature may be attributed to enhanced atomic mobility during growth, which facilitates nucleation and growth of the secondary phase. Therefore, the structural evolution observed in Figure 3 indicates a transition from predominantly substitutional incorporation at low dopant concentrations to phase segregation and Cu_7_Te_5_ formation at higher contents.

### Vibrational and structural surface properties

**Figure 4:**
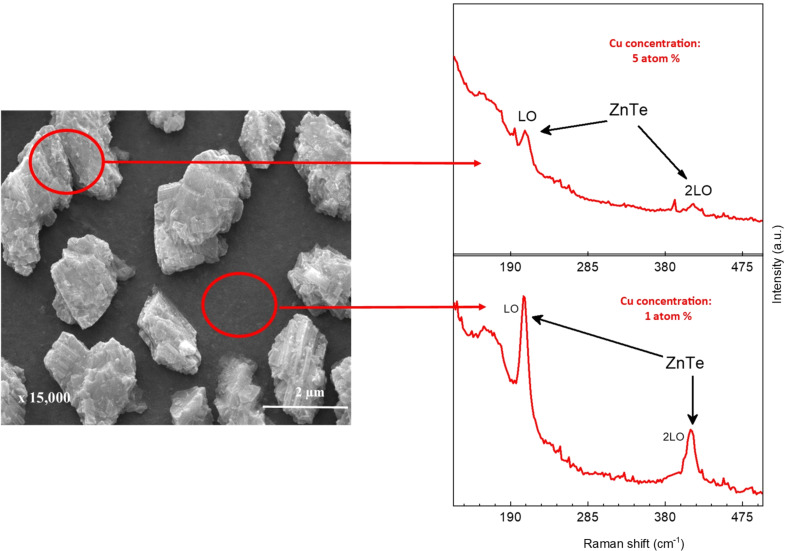
Surface micrograph and Raman spectra of CuZnTeO film with a nominal concentration of 3 atom % of Cu and O grown at 300 °C.

Figure 4 shows Raman spectra and a scanning electron microscopy (SEM) image of the CuZnTeO film grown at 300 °C with a nominal Cu and O concentration of 3 atom %. The SEM image reveals an irregular surface morphology characterized by agglomerate formation with non-uniform grain size distribution, suggesting localized compositional fluctuations during growth. According to the EDS analysis, two distinct compositional regions are identified, that is, a Cu-rich zone (≈5 atom %) and a Cu-poor zone (≈1 atom %).

Raman spectra collected from these regions exhibit vibrational bands associated with the longitudinal optical (LO) mode and its second-order overtone (2LO), characteristic of polycrystalline ZnTe. The ZnTe LO mode is observed at approximately 207 cm^−1^, while the 2LO mode appears near 413 cm^−1^, consistent with previous reports for ZnTe thin films. Notably, regions corresponding to the agglomerates display a significant reduction in the intensity of the LO and 2LO modes. This attenuation of ZnTe-related vibrational modes suggests the presence of secondary phases, most likely Cu_2−_*_x_*Te, which exhibit different vibrational signatures and reduced ZnTe phonon activity [28]. In contrast, the darker regions in the SEM image exhibit strong LO and 2LO Raman modes, indicating that these areas are predominantly composed of ZnTe. The spatial variation in Raman intensity supports the structural segregation observed by XRD, confirming the coexistence of ZnTe and Cu_2−_*_x_*Te phases within the film. The correlation between morphological irregularities, compositional inhomogeneity, and vibrational response indicates that even at relatively low Cu concentrations (3 atom %), local phase segregation occurs, leading to heterogeneous structural regions across the film surface.

**Figure 5:**
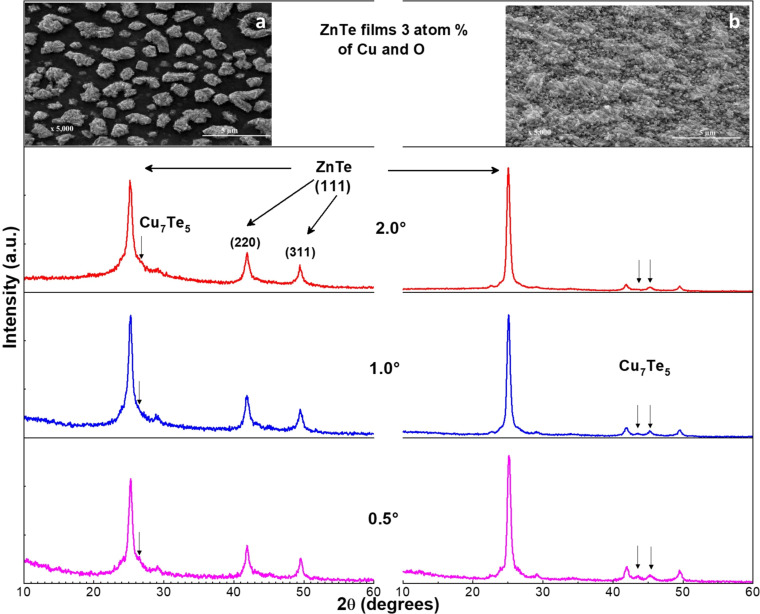
SEM images and X-ray diffraction of CuZnTeO films with nominal concentration of Cu and O of 3 atom % for substrate temperatures of (a) 300 °C and (b) 350 °C.

Figure 5 shows SEM images and grazing-incident X-ray diffraction (GIXRD) patterns of CuZnTeO films with a nominal Cu and O concentration of 3 atom % grown at substrate temperatures of 300 and 350 °C. The GIXRD measurements were performed at incidence angles of 0.5°, 1.0°, and 2.0°, corresponding to approximate X-ray penetration depths of 80, 160, and 320 nm, respectively. This angular variation allows for the evaluation of phase distribution as a function of film depth. The SEM images reveal morphological differences between films grown at 300 and 350 °C. At 300 °C, the surface exhibits well-defined agglomerated grains, whereas films grown at 350 °C display a more compact and continuous morphology, suggesting enhanced surface diffusion and grain coalescence at the higher substrate temperature.

In the GIXRD patterns, a mixture of cubic and hexagonal ZnTe phases can be identified at all incidence angles. In addition, diffraction peaks corresponding to the Cu_7_Te_5_ phase are detected. Importantly, the Cu_7_Te_5_ reflections are observed at all incidence angles, indicating that this secondary phase is not confined to the near-surface region but distributed throughout the probed film thickness. The relative intensity of Cu_7_Te_5_ peaks increases slightly with increasing incidence angle, suggesting a possible variation in volumetric fraction with depth. These results indicate that, even at a relatively low nominal Cu concentration (3 atom %), partial phase segregation occurs, leading to coexistence of ZnTe and Cu_7_Te_5_ phases across the film thickness. The presence of Cu_7_Te_5_ throughout the depth supports the compositional inhomogeneity observed by SEM and Raman analysis.

### Optical properties

**Figure 6:**
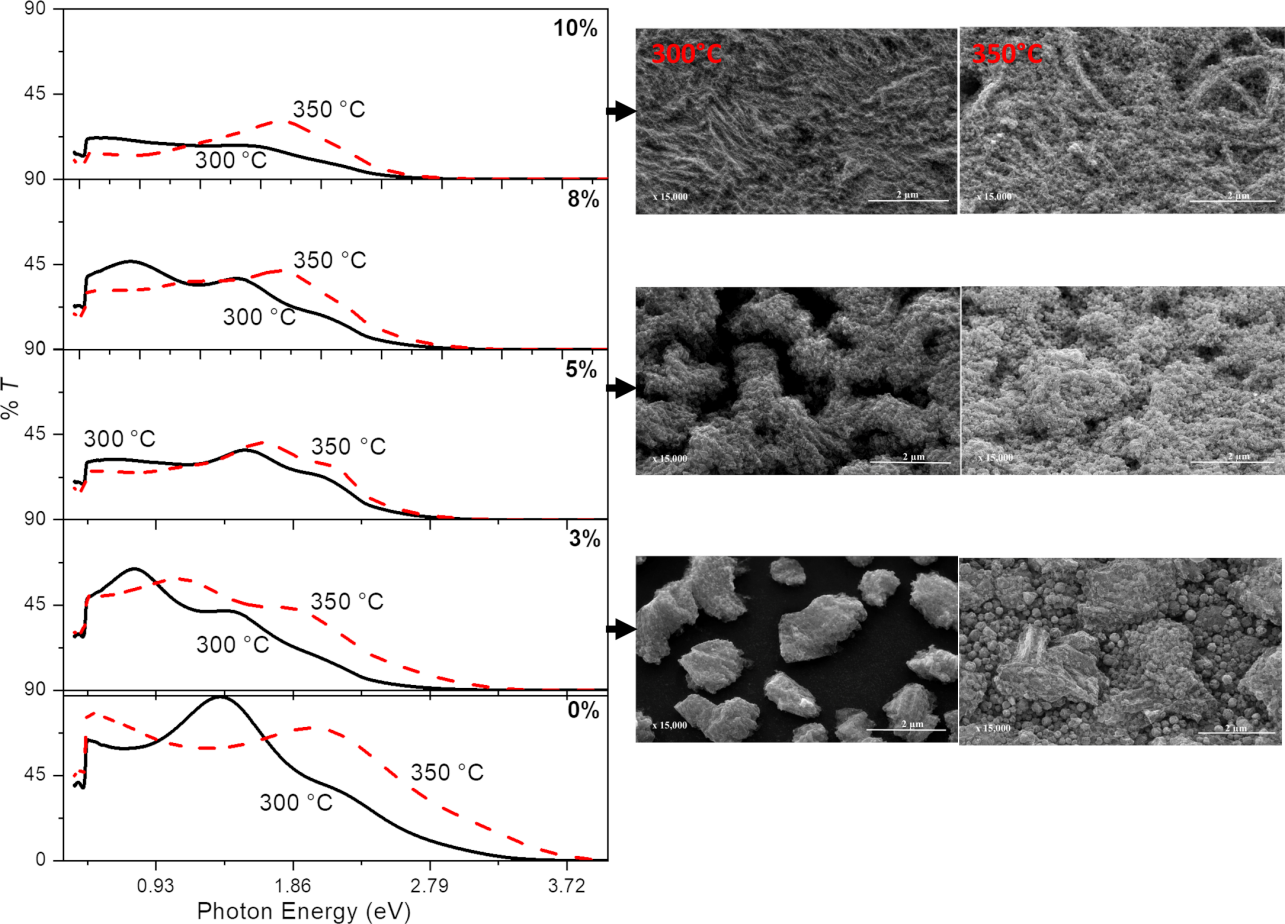
Transmittance spectra and SEM micrographs of CuZnTeO films grown on glass substrates as a function of Cu and O concentration for substrate temperatures of 300 °C (solid line) and 350 °C (dotted line).

The optical properties of the CuZnTeO films are strongly influenced by the nominal Cu and O concentration. Figure 6 shows the normal-incidence transmittance spectra of films grown on glass substrates at 300 and 350 °C. The optical bandgap was determined using Tauc method, considering a direct allowed transition, since ZnTe is a direct-bandgap semiconductor [38]. This procedure is commonly used from ZnTe and Cu-doped ZnTe thin films, as reported in previous studies [23–24]. The undoped ZnTe film was used as the reference system and exhibited an optical bandgap of approximately *E*_0_ ≈ 2.26 eV, consistent with reported values for RF-sputtered [26] and crystalline ZnTe thin films [39–41]. The optical bandgap, estimated from the Tauc analysis, remained close to the value measured for undoped ZnTe (≈2.26 eV) over the investigated composition range. Therefore, no significant bandgap modifications were observed as a function of Cu and O concentration. Consequently, the changes observed in the transmittance spectra are mainly attributed to enhanced absorption and light scattering phenomena resulting from the formation and growth of Cu-rich secondary phases rather than to significant modifications of the intrinsic band structure.

The undoped ZnTe film shows well-defined interference fringes in the photon energy region below the optical bandgap (*E*_0_ ≈ 2.26 eV), indicating good optical quality and relatively smooth film surfaces. Similar interference oscillations are observed for films containing 3–10 atom % Cu and O, suggesting that the films remain partially transparent and maintain a certain degree of thickness uniformity at low dopant concentrations.

However, in the infrared region, a significant reduction in transmittance is observed for Cu-containing films compared to pure ZnTe. This decrease in infrared transmission is attributed to the formation of Cu_2−_*_x_*Te phases, which exhibit high free-carrier absorption and metallic-like optical behavior in this spectral range [28]. As the Cu and O concentration increases, the films progressively acquire a darker appearance, indicating enhanced optical absorption across a broader energy range. This behavior is consistent with the structural results that reveal phase segregation and the presence of Cu-rich secondary phases.

Specular reflectance spectra could not be reliably measured due to the dark surface appearance and significant light scattering effects. The increased surface roughness and agglomerate formation observed in the SEM images (Figure 6) contribute to diffuse scattering, which reduces specular reflectance and alters the overall optical response of the films [9]. Overall, the optical response reflects a transition from semiconducting ZnTe-dominated behavior at low Cu concentration to increased absorption and reduced transparency associated with Cu_2−_*_x_*Te phase formation at higher dopant levels. The observed optical behavior is therefore mainly associated with Cu incorporation and the evolution of Cu-related secondary phases. Since no oxygen-related crystalline phases were detected and no distinct optical features could be directly attributed to oxygen incorporation, its contribution to the optical response could not be independently identified under the present experimental conditions.

### Electrical properties

The electrical transport properties of CuZnTeO thin films were evaluated by Hall effect measurements at room temperature. Silver and carbon contacts were deposited and verified to exhibit ohmic behavior prior to measurement. Film thickness was determined by profilometry and used for resistivity calculations.

**Figure 7:**
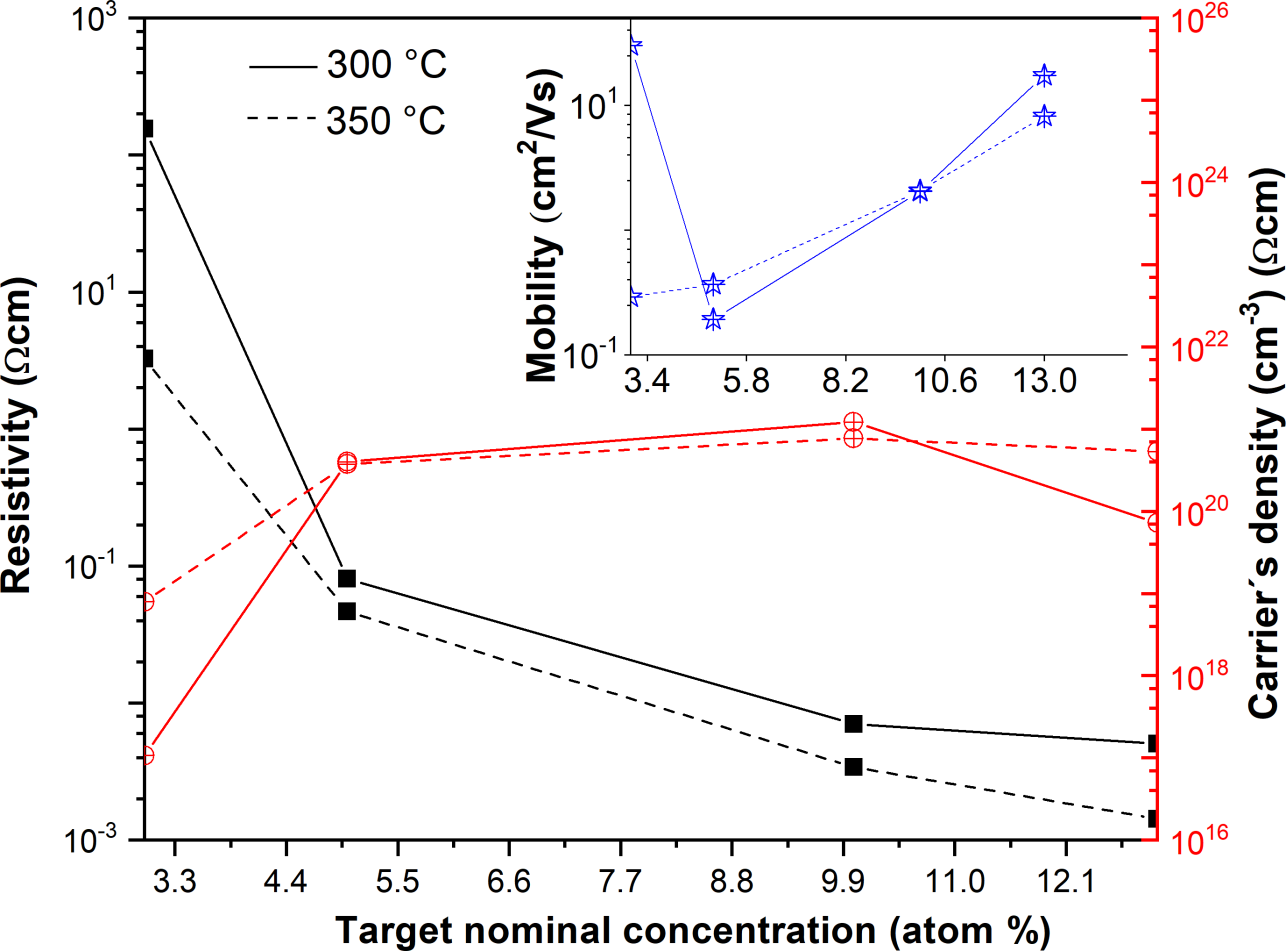
Electrical charge transport properties of CuZnTeO films grown on glass substrates as a function of nominal Cu and O concentration for substrate temperatures of 300 °C (continuous lines) and 350 °C (dotted lines).

Since the electrical transport properties of RF-sputtered undoped ZnTe are well established in the literature, they are not repeated here [42–43]. Figure 7 focuses on Cu-containing films (3–13 atom %), for which the influence of Cu incorporation and Cu_2−_*_x_*Te secondary-phase formation on the electrical transport properties can be directly evaluated. The figure presents the resistivity, carrier concentration, and mobility as a function of nominal Cu and O concentration for substrate temperatures of 300 °C (solid line) and 350 °C (dotted line). A clear decrease in resistivity is observed with increasing nominal Cu and O concentration. For films grown at 300 °C, the resistivity decreases from approximately 10^2^ Ω·cm at 3 atom % to about 10^−2^ Ω·cm at 13 atom %. Similarly, films grown at 350 °C exhibit a reduction from approximately 10^1^ Ω·cm (3 atom %) to 10^−3^ Ω·cm (13 atom %).

This several-orders-of-magnitude reduction in resistivity indicates a transition from semiconducting to highly conductive behavior with increasing Cu and O incorporation. The decrease in resistivity is attributed to the progressive formation of Cu_2−_*_x_*Te-related phases, which exhibit degenerate p-type or metallic-like conductivity.

The lower resistivity observed at 350 °C compared to 300 °C for the same nominal concentration suggests that higher substrate temperature promotes enhanced formation and growth of Cu_7_Te_5_ secondary phases, consistent with the XRD and GIXRD results. Increased atomic mobility at higher temperatures likely facilitates Cu diffusion and phase segregation, resulting in a larger volumetric fraction of highly conductive Cu-rich regions. The increase in carrier concentration and conductivity is consistent with the role of Cu as an acceptor dopant in ZnTe. In contrast, no direct evidence for a significant contribution of oxygen to the electrical transport properties was obtained under the present experimental conditions. This observation is consistent with the structural results, which show that the evolution of the films is primarily governed by Cu incorporation and the formation of Cu-rich secondary phases.

At the highest Cu and O concentration (13 atom %), a deviation from the general trend is observed in the carrier concentration. This behavior is likely associated with the onset of phase segregation and microstructural evolution, as suggested by the XRD results, which reveal the formation and growth of Cu_7_Te_5_ secondary phases at high Cu contents. Under these conditions, the electrical response is no longer governed exclusively by the ZnTe matrix, but also by conductive Cu-rich regions that contribute increasingly to carrier transport. Similar behavior has been reported in Cu-containing telluride systems, where increasing Cu concentration promotes secondary phase formation and significant modifications of the electrical response [27–28].

Therefore, the observed deviation may result from the combined effects of limited Cu solubility in ZnTe, the increasing volume fraction of conductive Cu-rich phases, and the associated microstructural evolution. The simultaneous increase in both carrier concentration and mobility suggests that electrical transport becomes dominated by highly conductive Cu_2−_*_x_*Te clusters rather than solely by substitutional doping within the ZnTe lattice. The increasing fraction of these secondary phases facilitates carrier transport through the film, resulting in a strong decrease in resistivity. This interpretation is consistent with the XRD and GIXRD results, which reveal the formation and growth of Cu_7_Te_5_-related phases, as well as with the microstructural evolution observed by SEM [27–28]. Overall, the electrical results corroborate the structural and vibrational analyses, confirming that increasing Cu and O concentration induces a transition from predominantly ZnTe semiconducting behavior to a composite system dominated by conductive Cu_2−_*_x_*Te phases.

## Conclusion

The growth of CuZnTeO thin films from a single target of ZnTe-CuO composite target by RF magnetron sputtering was systematically investigated. The results demonstrate that the film composition and resulting phase evolution can be controlled by varying the nominal target composition and substrate temperature. Structural analysis revealed the coexistence of polycrystalline ZnTe and Cu_2−_*_x_*Te phases. No evidence of metallic Cu was detected. Increasing substrate temperature and dopant concentration promotes enhanced crystallinity of ZnTe as well as the formation and growth of Cu_2−_*_x_*Te secondary phases. Raman spectroscopy and SEM images confirmed that the films exhibit a composite microstructure consisting of ZnTe and Cu_2−_*_x_*Te regions, in agreement with XRD and GIXRD results. The reduction in optical transmittance in the infrared region correlates with the presence of Cu_2−_*_x_*Te phases, which exhibit metallic-like absorption behavior. Electrical measurements revealed a several-orders-of-magnitude decrease in resistivity with increasing Cu and O concentration, accompanied by significant increases in carrier concentration and mobility. These results indicate a transition from semiconducting ZnTe-dominated transport to conduction governed by conductive Cu-rich phases, associated with their increasing volume fraction and the resulting microstructural evolution.

Overall, the studied system behaves as a tunable composite material whose structural and electrical property can be engineered via controlled Cu and O incorporation. The obtained conductivity levels and phase stability indicate that these films behave as a biphasic system composed of semiconducting ZnTe and conductive Cu-rich telluride phases. Although no device-level validation was performed, the observed structure-property relationships provide useful insight for the future development of ZnTe-derived materials intended for back-contact applications in CdTe solar cells.

## Data Availability

Data generated and analyzed during this study is available from the corresponding author upon reasonable request.
